# Obstetric Society of Philadelphia

**Published:** 1883-10-20

**Authors:** 


### Southern Medical Record:

EDITORS:

T. S. POWELL, M.D. W. T. GOLDSMITH, M.D. R. C. WORD, M.D.

*R. C. WORD, M.D., Managing Editor.*

☞All Communications and Letters on Business connected with the RECORD must be addressed to the Managing Editor.

VOL. XIII. ATLANTA, GA., OCTOBER 20, 1883. No. 10.

### ORIGINAL AND SELECTED ARTICLES.

### OBSTETRIC SOCIETY OF PHILADELPHIA.

A stated meeting was held September 6, 1883, the President, R. A. Cleemann, M. D., in the chair.

*Face Presentation with Eclampsia.*—Dr. William T. Taylor read the report of a case, as follows:

"Face presentations are somewhat rare. Dr. Churchill said, some years ago, in recording the statistics found, that in British practice they occurred once in 292 cases; in French practice once in 275 cases; and in German practice, once in 130 cases. In my own practice I have met with about one dozen, and as the last one was combined with eclampsia, I will report it to the society.

"During utero-gestation my patient enjoyed very good health, having no headache, no swollen limbs nor bloated features, no vertigo or dimness of vision. There was no deficiency of urine, and therefore I did not examine it for albumen. Her appetite was fair, her bowels were regular, and she took a moderate degree of exercise; so I had no reason to expect trouble when labor began.

On May 14, 1883, I was summoned, at 6 a. m., to visit Mrs. C. Haley, aged 23 years, a primipara, who was in the first stage of labor, having had a show since midnight. On examination, I found the os very slightly dilated, with the pains "few and far between," and the face of the child presenting, with the chin toward the sacrum. The nurse informed me that the patient had not slept during the night, and was very nervous and irritable. Her skin was moist, her pulse was normal, and she had urinated frequently. I gave her a mixture containing hydrate of chloral, bromide of potassium, and valerianate of ammonium, to compose her, and went home to my breakfast, intending to return in a few hours. At eight o'clock her husband came to my office and told me that his wife 'had had a fit, and could not keep the medicine down.' I arrived at the house at 8:30 a. m., and sent immediately for some powdered hydrate of chloral and an injection apparatus. The patient had had two convulsions, which were ushered in by complainings of her head, her face being very red, and her head drawn to one side, with the features much distorted. The first convulsion occurred when the nurse was about to give the first dose of the medicine. Directly after my arrival a third convulsion occurred, and lasted for a minute or more, her head being violently drawn to the right side, with jerking of her arms and legs. I dissolved one drachm of the hydrate chloral in about four ounces of water, and threw it into the rectum. The fit yielded immediately. As she was unconscious, I had an excellent opportunity of examining her. The os was dilated to the size of a quarter of a dollar, and soft, so that it yielded gradually to the pressure of my fingers, when I discovered the face presentation, with the chin toward the left sacro-iliac junction. I endeavored to push the chin toward the breast, so as to bring down the occiput in the second position of Baudelocque. This I found somewhat difficult; but, as the os dilated under the pressure of my fingers, I reached the occiput, and, after several attempts, succeeded in bringing it down to a favorable position—the one aforesaid. My patient, by this time, was becoming restless and uncontrollable, and, fearing another convulsion, I again gave her an injection of chloral, which quieted her. Having placed her on her back, and brought her to the edge of the bed, her limbs being supported by the nurse and another woman, the forceps was easily applied, and the head brought down below the inferior strait. I removed the instrument when the head pressed against the perineum, allowing nature to finish the delivery.

The child, a boy, was still-born, the cord being pulseless. In fact, I was apprised of this while endeavoring to dilate the os with my fingers, for a significant tremor had passed through the body of the child, assuring me of its death. The placenta was removed quite easily.

"During all this time my patient was unconscious, and had no return of convulsions from the time I gave her the first injection of chloral. As her pulse was good and her respiration easy, I applied a binder, and, having placed her in a comfortable position, left her sleeping.

On my return, at 5 p. m., she was restless and slightly feverish, but, after taking a few doses of chloral and valerian, she was quieted to sleep. On the next morning, May 15th, she was perfectly conscious; pulse 80, temperature 99° F., and respiration normal. She had urinated freely, and, with the exception of slight soreness over the abdomen, was very comfortable. She inquired for the baby, knowing from her condition that it had been born, but the preceding twenty-four hours were to here a perfect blank.

From this time she had no further trouble, and soon recovered. This case certainly showed the beneficiel effect of injections of hydrate of chloral in controlling puerperal convulsions when they are of a nervous form."

Dr Albert H. Smith remarked that face presentations and puerperal convulsions presented a large field for discussion. Dr. Taylor was very fortunate to be able to bring down the occiput and keep it down until the forceps could be applied. In this operation a man needed three hands—one to hold the head while the others manipulated the instrument. The mechanism of a primary face-presentation, as reported in this case, was difficult to understand. It might occur secondarily from obliquity of the uterus and a sudden rush of waters, causing a sudden engagement of the head before flexion could be secured. In such cases it was very difficult to secure and maintain flexion until the forceps could be applied. In the majority of cases of face presentation, even with the chin posteriorly, nature was best able to terminate the case satisfactorily. It was to this class that the aphorism "meddlesome midwifery is bad" was most applicable. The natural forces work slowly, and the neck of the child became accustomed to the extreme extension which it had to undergo, while it was very bad to bring a sudden strain on the vertebræ and other tissues of the neck by too rapid forcing of the chin into violent extension by means of the forceps or otherwise. The consequence of the hasty proceeding was a still-born child. The only ground for interference was an alarming condition of the child's pulse. If the child's heart was beginning to fail, we must take the risk and give it the benefit of the chance. The child's head could not be born in a face presentation until the chin had engaged under the pubes. The old teaching was that the chin posterior could not be born ; but he was very early undeceived on this point, one of his earliest cases having been of that character. He had sent for his preceptor to come and bring perforating instruments, but, while awaiting their arrival, nature proved equal to the task, rotation occurred spontaneously, and a living child was born."

Dr. B. F. Baer inquired if version by the feet would not be much preferable to waiting for nature to deliver in chin-posterior positions ?

Dr. Smith did not mean that we should never interfere in a case of this kind, but that a large majority, if left to nature, would terminate spontaneously by anterior rotation of the chin, with safety to both mother and child. He would decidedly negative the proposition of version by the feet, because, the amniotic sac having been necessarily ruptured by previous efforts to bring down the vertex, the waters would have been completely evacuated, and the uterus would be in a condition of spasmodic contraction, so that an attempt to turn would involve great danger of rupture of the organ. The introduction of the hand always increased the risk of septic absorption—two terrible risks for the mother, while the child was exposed to all the dangers of head-last delivery. He would consider chin posterior presentations natural labors, and would allow them to terminate spontaneously unless there was some complication demanding version.

Dr. J. G. Allen coincided with Dr. Smith in his conservative principles. The risks of version to the mother were great—too great to allow it to be performed for the sake of the child. The operation of version was not looked upon in so serious a light as it should be, under all circumstances. In some instances it might be very easy, and might terminate well, but in others, apparently similar in conditions, the results to the mothers were bad. He would not lose one mother to save ten children. He would never resort to version unless the labor was impossible under other measures. Even after it was skillfully performed the child was often still-born. The increased risk to the mother was followed by no corresponding gain in safety to the child.

Dr. R. P. Harris thought the ideas of Dr. Smith were the same as held by most eminent obstetricians, and agreed with their practice as expressed to him in private correspondence.

Dr. Baer was willing to be taught. The views expressed this evening did not harmonize with the teaching of even the present day in Philadelphia. He had been taught that version would be proper if the case was diagnosticated early and the operation could be performed before the waters were evacuated, and it seemed to him that the rational thing under such circumstances would be to turn. It was entirely a new light to him to consider chin-posterior cases as easy, natural labor. He had been taught to look upon them as impossible of spontaneous completion, rotation never taking place, the forces in action not being great enough to compel it. His own recent experience had led him to doubt this dictum; with one blade of the forceps, used as a vectis, he had without difficulty secured anterior rotation. His idea of the impossibility of rotation under the circumstances had made him doubt the correctness of his diagnosis of the position, but the principles put forth this evening reassured him. Might the death of the child, causing relaxation, be the cause of the face presentation?

Dr. Allen did not expect others to accept his opinion, but in his denunciation of turning he alluded to the complete transposition of one extremity of the fœtal ellipse for the other, and did not include the changing of one part of the head for another; but, in the first class, the poor chance of saving the child would not compensate for the increased danger to the mother.

Dr. Smith did not consider the chin-posterior an easy natural labor. On the contrary, it was the most difficult of natural labors. The chin struck upon the posterior inclined planes and was rotated to an anterior position, in which it engaged under the arch of the pubes exactly as the vertex would. In multiparæ, nature was able to accomplish this result, but in primiparæ assistance in rotation might be required, and even traction might become necessary. In contrasting the dangers incident to verson by the feet and those involved in trusting to nature in this condition when the waters had been discharged, as they necessarily had, in the attempts to bring down the vertex, which would be first tried, we must remember that the child would be tightly grasped by the uterus, and that it must be twisted upon its long axis as well as turned to bring the nape of the neck under the arch of the pubes, and that this procedure would greatly enhance the danger to both mother and child.

Dr. Taylor, in closing the discussion, remarked that the death of the child occurred after it was fully engaged, and was not a factor in causing the face presentation. When he made his diagnosis of position, the head was high up, and, the child being small, he had no difficulty in bringing the vertex down.—*N. Y. Med. Record.*

